# Efficient genetic manipulation of *Shewanella* through targeting defense islands

**DOI:** 10.1128/aem.02499-24

**Published:** 2025-03-21

**Authors:** Yilong Ruan, Huan Tang, Tongxuan Cai, Xiaofei Du, Tianlang Liu, Xiaoxue Wang, Pengxia Wang

**Affiliations:** 1 Key Laboratory of Tropical Marine Bio-resources and Ecology, South China Sea Institute of Oceanology, Chinese Academy of Sciences 655251 , Guangzhou, China; 2 Key Laboratory of Tropical Oceanography, South China Sea Institute of Oceanology, Chinese Academy of Sciences, Guangzhou, China 606379 , Guangzhou, China; 3 University of Chinese Academy of Sciences 74519, Beijing, China; 4 College of Life Sciences, Hebei Normal University 117828, Shijiazhuang, Hebei, China; University of Nebraska-Lincoln, Lincoln, Nebraska, USA

**Keywords:** *Shewanella*, *Shewanella putrefaciens*, genetic manipulation, defense system, genomic islands

## Abstract

The *Shewanella* genus is widely recognized for its remarkable respiratory adaptability in anaerobic environments, exhibiting potential for bioremediation and microbial fuel cell applications. However, the genetic manipulation of certain *Shewanella* strains is hindered by defense systems that limit their genetic modification in biotechnology processes. In this study, we present a systematic method for predicting, mapping, and functionally analyzing defense islands within bacterial genomes. We investigated the genetically recalcitrant strain *Shewanella putrefaciens* CN32 and identified several defense systems located on two genomic islands integrated within the conserved chromosomal genes *trmA* and *trmE*. Our experimental assays demonstrated that overexpression of excisionases facilitated the excision of these islands from the chromosome, and their removal significantly enhanced the genetic manipulation efficiency of *S. putrefaciens* CN32. Further analysis revealed that these defense islands are widespread across various *Shewanella* strains and other gram-negative bacteria. This study presents an effective strategy to circumvent genetic barriers and fully exploit the potential of *Shewanella* for environmental and microbial engineering applications.

**IMPORTANCE:**

Efficiently modifying bacterial genomes is critical for advancing their industrial applications. However, bacteria in complex environments naturally develop defense mechanisms in response to bacteriophages and exogenous DNA, which pose significant challenges to their genetic modification. Several methods have emerged to tackle these challenges, including *in vitro* methylation of plasmid DNA and targeting specific restriction-modification (R-M) and CRISPR loci. Nevertheless, many bacteria harbor multiple, often uncharacterized defense mechanisms, limiting these strategies. Our study differs from previous approaches by specifically targeting defense islands–clusters of defense systems located within mobile genetic elements. Here, we investigated *Shewanella putrefaciens* CN32 and identified two key defense islands responsible for these protective functions. By selectively deleting these defense islands, we significantly enhanced the efficiency of genetic manipulation in *S. putrefaciens*. Our findings not only demonstrate a promising strategy for improving genetic engineering in *Shewanella* but also suggest broader applicability across other bacterial species. This work opens new opportunities for optimizing microbial processes in biotechnology, highlighting the potential of defense island-targeted genetic modification.

## INTRODUCTION

Bacteria belonging to the *Shewanella* genus are widely known for their extraordinary respiratory versatility, particularly under anaerobic conditions. This adaptability allows them to utilize a wide range of organic and inorganic compounds as terminal electron acceptors, playing a significant role in biogeochemical cycling processes (1). Most notably, *Shewanella* species can reduce metal ions such as Fe(III) and Mn(IV) under anaerobic conditions, enabling them to survive and thrive in environments where oxygen is scarce (2, 3). The diverse metabolic capabilities of *Shewanella* play a crucial role in biogeochemical cycling processes, and they hold significant potential for bioremediation of heavy metal contamination and applications in microbial fuel cells (4).

One of the most studied species, *Shewanella putrefaciens*, is a model organism for dissimilatory iron reduction, using various metal ions as terminal electron acceptors (1, 2, 5). This flexibility allows it to thrive in diverse habitats, with strains such as *S. putrefaciens* CN32, W3-18-1, 200, and ATCC 8071 isolated from environments ranging from anaerobic shale sandstone to deep marine sediments, a Canadian oil pipeline, and butter in England (1). The respiratory flexibility of *S. putrefaciens* has drawn attention to its unique physiological traits, which are the subject of extensive studies focused on signal transduction pathways, physiology, and metabolic regulation (6, 7). These insights are invaluable for better understanding the metabolic versatility of this genus and its potential applications.

One promising area of research involves the genetic engineering of *Shewanella* species to enhance their ability to degrade specific contaminants, boost bioenergy production, or optimize their application in microbial fuel cells. By manipulating their metabolic pathways, researchers can increase their efficiency in bioenergy production, making these organisms valuable for renewable energy applications (8, 9). However, the genetic modification of *Shewanella* can be challenging due to the presence of defense mechanisms, such as restriction-modification (R-M) systems and clustered regularly interspaced short palindromic repeat (CRISPR) systems (10). To overcome these challenges, the deletion of defense systems has been employed to facilitate more efficient genetic modifications. For instance, the strain *S. putrefaciens* W3-18-1 harbors a restriction-modification system, PstI/PstM, encoded within a genomic island (SGI1Sp), which poses a significant obstacle to genetic engineering. The knockout of the PstI/PstM system has been shown to enhance its genetic manipulability (11, 12).

Comparative analyses reveal that multiple defense systems are often clustered on defense islands, exhibiting considerable diversity (12, 13). Thus, targeting the removal of these defense islands may provide a more efficient and time-saving approach for bacterial genetic manipulation. In this study, we present a systematic method for predicting, mapping, and functionally analyzing defense islands within bacterial genomes. Our findings indicate that targeting these defense islands represents an effective strategy for improving bacterial genetic manipulation and opens new avenues for optimizing microbial processes in biotechnology.

## RESULTS

### Genomic islands integrated in *trmA* and *trmE* genes encoding multiple defense systems were identified in CN32

**Fig 1:**
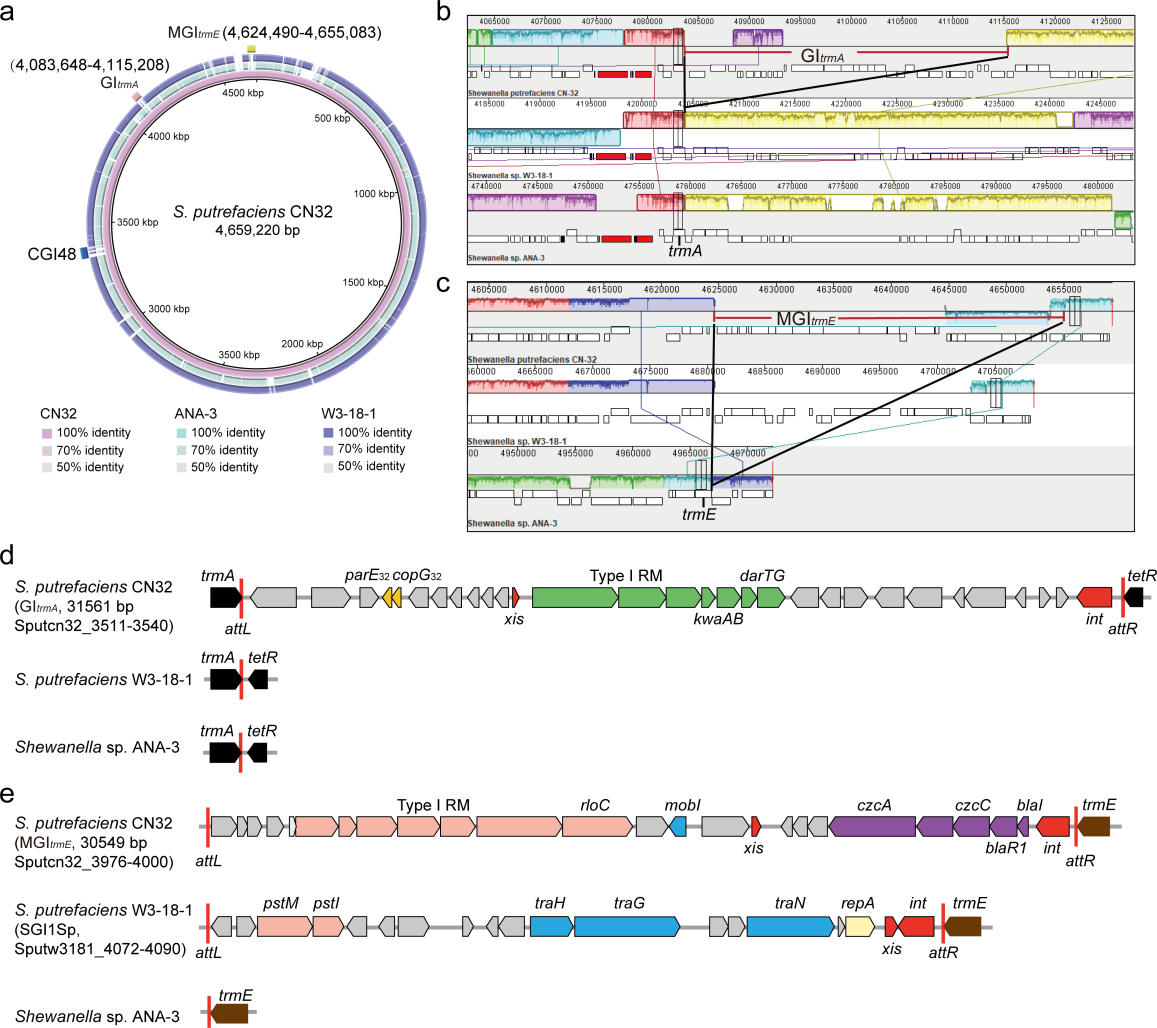
Three defense islands in *S. putrefaciens* CN32. (a) Genomic comparison of CN32 (CP000681) with W3-18-1 (CP000503) and ANA-3 (NC_008577) using BRIG showing the locations of the defense islands CGI48, GI*_trmA_*, and MGI*_trmE_* on the circular genome. Sequence-based synteny analysis of the CN32, W3-18-1, and ANA-3 genomes using Mauve to locate the positions of the two ends of GI*_trmA_* (b) and MGI*_trmE_* (c). The integration locus *trmA* and *trmE* were indicated. Mapping of the integration locus of GI*_trmA_* (d) and MGI*_trmE_* (e) in CN32, W3-18-1, and ANA-3. Open reading frames with putative functions are shown in different colors.

*S. putrefaciens* CN32 possesses a genomic size of 4,659 kb, comprising a single, circular chromosome with approximately 3,972 open reading frames (ORFs). Using DefenseFinder (14), 17 defense systems were identified in CN32, including R-M systems, dGTPase, CRISPR/Cas system, Kiwa, a DarTG, a Retron, a RloC, and other systems (Table 1). Defense systems are often clustered within genomic islands (15), promoting our investigation into the genomic islands of CN32. This was achieved by comparing the chromosomes of CN32 with those of related species, such as *S. putrefaciens* W3-18-1 and *Shewanella* sp. ANA-3 (Fig. 1a). The analysis revealed three specific genomic islands, CGI48, GI*_trmA_*, and MGI*_trmE_*, that encode multiple defense systems. CGI48, a composite genomic island, carries a 21 kb genomic island (GI21) (13) that includes genes encoding RM_Type_I and RM_Type_IV (Table 1).

**TABLE 1:** Defense systems in CN32 predicted using DefenseFinder

Number	Type	Subtype	Proteins in system	MGEs
1	RM	RM_Type_I	Sputcn32_0154, 0157, 0160–0162	
2	dGTPase	dGTPase	Sputcn32_0159	
3	PfiAT	PfiAT	Sputcn32_0176–0177	
4	AVAST	AVAST_V	Sputcn32_0703	
5	Cas	CAS_Class1-Subtype-I-F	Sputcn32_1819–1823	
6	dGTPase	dGTPase	Sputcn32_1870	
7	Retron	Retron_I_B	Sputcn32_2610–2611	
8	RM	RM_Type_I	Sputcn32_2908, 2909, and 2912	CGI48
9	RM	RM_Type_IV	Sputcn32_2910–2911	CGI48
10	Rst_PARIS	PARIS_I	Sputcn32_3206–3207	
11	RM	RM_Type_I	Sputcn32_3523–3525	GI*_trmA_*
12	Kiwa	Kiwa	Sputcn32_3526–3527	GI*_trmA_*
13	DarTG	DarTG	Sputcn32_3528–3529	GI*_trmA_*
14	Lamassu-Fam	Lamassu-Fam	Sputcn32_3840–3843	
15	PD-T4-5	PD-T4-5	Sputcn32_3948	
16	RM	RM_Type_I	Sputcn32_3981, 3884–3986	MGI*_trmE_*
17	RloC	RloC	Sputcn32_3987	MGI*_trmE_*

Furthermore, two additional genomic islands, GI*_trmA_* and MGI*_trmE_*, have been identified. Sequence-based synteny analysis of the CN32, W3-18-1, and ANA-3 genomes, conducted using the Mauve alignment tool, predicted the locations of the two ends of GI*_trmA_* and MGI*_trmE_* (Fig. 1b and c). Further investigations revealed that GI*_trmA_* is integrated within the *trmA* gene, responsible for encoding tRNA (uridine(54)-C5)-methyltransferase. It is 31,561 bp in length encoding three predicted defense systems, an RM_Type_I, a Kiwa, and a DarTG system. In contrast, W3-18-1 and ANA-3 lack genomic islands integrated within their *trmA* genes (Fig. 1d; Table S1). MGI*_trmE_* is inserted within the *trmE* gene, which encodes tRNA modification GTPase. It is 30,569 bp in length, which encodes two defense systems, an RM_Type_I system, a RolC protein, and five proteins associated with cobalt-zinc-cadmium resistance. Notably, W3-18-1 harbors an SGI1-like genomic island (SGI1Sp) within its *trmE* gene, whereas ANA-3 does not have any genomic islands integrated into its *trmE* gene (Fig. 1e; Table S2). Unlike SGI1Sp, which encodes *traH*, *traG*, and *traN*, MGI*_trmE_* encodes a conjugative transfer protein homologous to MobI(A/C), exhibiting 64% identity with the MobI found in MGI*Vch*Hai6. This protein is involved in the transfer of the genomic island in the presence of an IncC plasmid (16). This study underscores the presence of genomic islands integrated in *trmA* and *trmE* genes encoding multiple defense systems specifically in CN32.

### GI*_trmA_* and MGI*_trmE_* can excise from the CN32 chromosome

**Fig 2:**
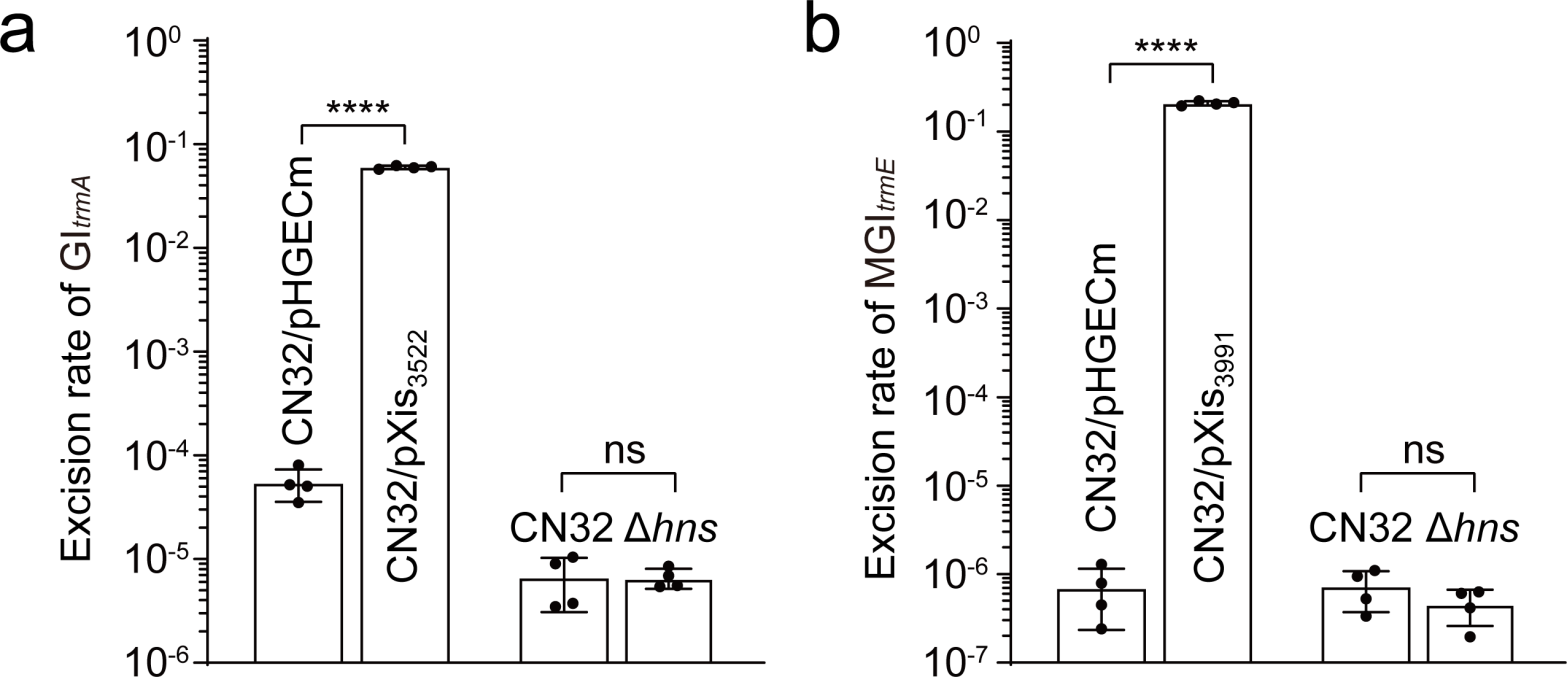
The excision of GI*_trmA_* and MGI*_trmE_* in CN32. The excision rate of GI*_trmA_* (a) and MGI*_trmE_* (b) when the corresponding excisionase Xis was overexpressed or when the *hns* gene was deleted in CN32 . The data are shown as the mean ± SD. For biological replicates, *n* = 4. Statistical analysis was performed using an unpaired *t*-test, “ns” denotes no significant difference, **** indicates *P* < 0.0001.

Sequence analysis revealed the presence of excisionases, Sputcn32_3522 (Xis_3522_) and Sputcn32_3991 (Xis_3991_), in GI*_trmA_* and MGI*_trmE_*, respectively. These proteins share conserved domains with the AlpA family excisionase of prophage CP4-57 regulatory protein. To determine the functionality of these excisionases and their role in promoting the excision of the corresponding genomic islands, experiments were conducted. Overexpression of Xis_3522_ led to a 1.1 × 10^3^-fold increase in the excision rate of GI*_trmA_*, reaching 6.0% (Fig. 2a). Similarly, overexpression of Xis_3991_ resulted in a 3.0 × 10^5^-fold increase in the excision rate of MGI*_trmE_*, reaching 20.8% (Fig. 2b).

Previous studies have shown that the host nucleoid structuring protein (H-NS) can regulate the excision of mobile genetic elements like prophage CP4So in *Shewanella oneidensis* (17). Therefore, the role of H-NS in regulating the excision of GI*_trmA_* and MGI*_trmE_* in CN32 was investigated using an *hns* deletion mutant. The results indicated that there was no significant difference in the excision rate of GI*_trmA_* and MGI*_trmE_* when *hns* was deleted (Fig. 2), suggesting that H-NS does not regulate the excision of these genomic islands in CN32.

### Construction of genomic island deletion mutants ΔGI*_trmA_* and ΔMGI*_trmE_*

**Fig 3:**
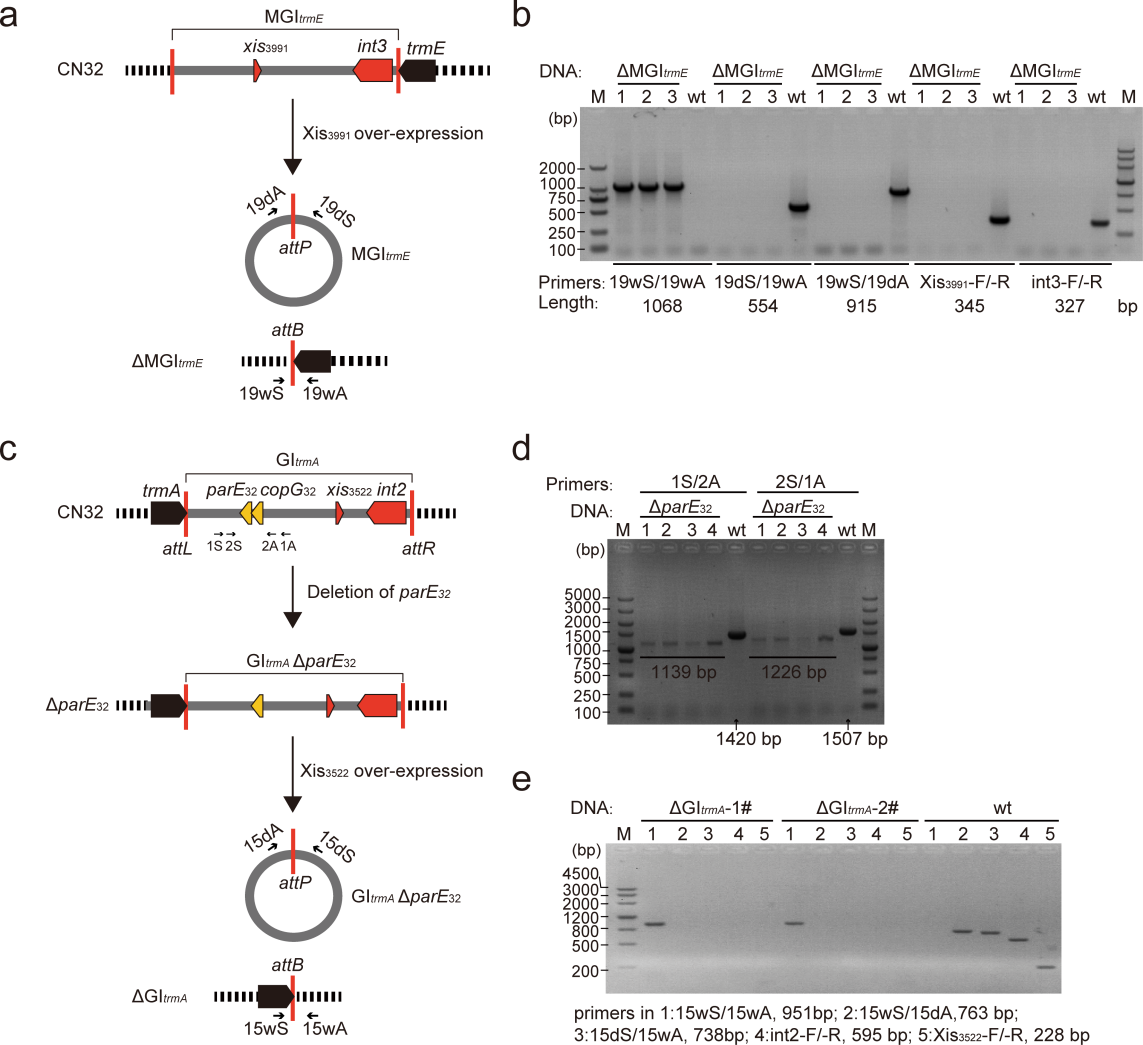
Construction and verification of mutant strains ΔGI*_trmA_* and ΔMGI*_trmE_*. (a) A schematic diagram illustrating the construction of ΔMGI*_trmE_* using Xis_3991_ over-expression. (b) PCR verification of ΔMGI*_trmE_*. (c) A schematic diagram illustrating the construction of ΔGI*_trmA_* using Xis_3522_ over-expression in the toxin gene deletion mutant Δ*parE*_32_. (d) PCR verification of Δ*parE*_32_ using the indicated primers in c. (e) PCR verification of ΔGI*_trmA_*.

To explore the roles of GI*_trmA_* and MGI*_trmE_* in host bacteria, we constructed deletion mutants of these mobile genetic elements. By employing an excisionase-based method for the removal of genomic islands (12, 17, 18), we aimed to generate the ΔGI*_trmA_* and ΔMGI*_trmE_* mutants using Xis_3522_ and Xis_3991_ overexpression in CN32 (Fig. 3). Our findings revealed that obtaining the ΔMGI*_trmE_* mutant was straightforward with Xis_3991_ overexpression in CN32 (Fig. 3a and b). However, acquiring the ΔGI*_trmA_* mutant proved challenging when Xis_3522_ was overexpressed in CN32.

Previous studies have highlighted the critical role of toxin-antitoxin (TA) systems encoded by genomic islands or prophages in stabilizing these elements after excision. For example, the ParEso/CopAso system in the CP4So prophage of *Shewanella oneidensis* MR-1 and the HipA/HipB system in CGI48 of CN32 do not directly affect the excision process but contribute to the maintenance of these elements in host cells post-excision (13, 19). TA systems consist of a toxin and its cognate antitoxin. The toxin is toxic to bacterial cells, whereas the antitoxin counteracts this toxicity. TA systems stabilize genomic islands or prophages through a mechanism known as postsegregational killing (PSK), ensuring the maintenance of these elements by killing cells that lose them, thereby creating an “addiction” to the element within the bacterial population (13, 19). In this study, we hypothesized that the putative TA system Sputcn32_3515-Sputcn32_3514 might maintain GI*_trmA_* after excision, thus hindering the generation of GI*_trmA_* deletion mutant.

To investigate this, we assessed the functionality of Sputcn32_3515 and Sputcn32_3514 as a TA pair. Sequence analysis showed Sputcn32_3515 (designated CopG_32_) encodes a 94 aa transcriptional regulator with 92% identity to CopAso, whereas Sputcn32_3514 (designated ParE_32_) encodes a 97 aa protein with 99% identity to ParEso (Fig. S1a). To assess the functionality of this putative TA pair, we cloned the genes into pCA24N-based plasmids and tested their expression in *E. coli* BW25113 cells. We found that the overproduction of ParE_32_ induced toxicity, whereas CopG_32_ did not exhibit toxicity and effectively counteracted the toxicity of ParE_32_ (Fig. S1b through e). These results confirmed that ParE_32_ and CopG_32_ form a functional TA system, with ParE_32_ contributing to the maintenance of GI*_trmA_* after excision.

Building on these findings, we disrupted the toxin gene *parE_32_* within GI*_trmA_* in CN32, generating the Δ*parE_32_* strain (Fig. 3c and d). The ΔGI*_trmA_* mutant was successfully generated in the Δ*parE_32_* strain by overexpressing the excisionase Xis_3522_ (Fig. 3e). These results suggest that the TA system ParE_32_/CopG_32_ plays a key role in maintaining GI*_trmA_* post-excision, complicating the generation of knockout mutants of GI*_trmA_* in the presence of this TA system.

### GI*_trmA_* and MGI*_trmE_* can help bacterial host defense against phages and plasmids

**Fig 4:**
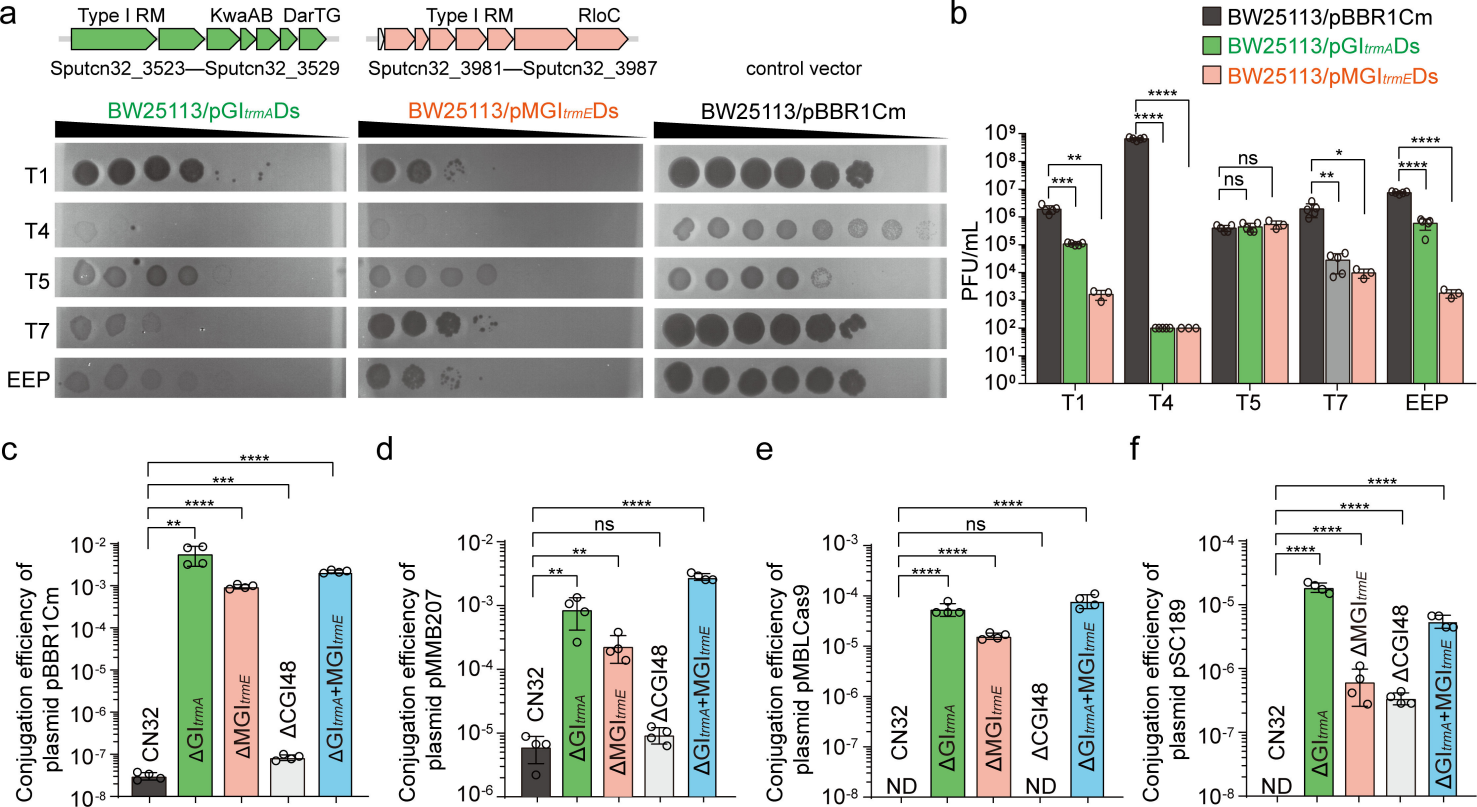
GI*_trmA_* and MGI*_trmE_* can help host bacteria defense against phages and plasmids. Representative images (a) and the quantification (b) of the efficiency of plating (EOP) of phages T1, T4, T5, T7, and EEP infection in engineered strain BW25113/pGI*_trmA_*Ds (*n* = 5), BW25113/pMGI*_trmE_*Ds (*n* = 3), and control strain BW25113/pBBR1Cm (*n* = 5). The defense systems in GI*_trmA_* and MGI*_trmE_* with locus tag accessions cloned in the engineered bacteria are indicated. (c-f) Conjugation efficiency of plasmid pBBR1Cm, pMMD207, pMBLCas9, and pSC189 to strains ΔGI*_trmA_* and ΔMGI*_trmE_*, compared with CN32 wild type. “ND” stands for “not detectable,” indicating that the conjugation efficiency for samples CN32 and ΔCGI48 in panels e and f was below the limit of detection (1 × 10^−8^). For statistical analysis, the value of 1 × 10^−8^ was used. The data are shown as the mean ± SD. For biological replicates: panels c-f, *n* = 4. Statistical analysis was performed using an unpaired *t*-test, “ns” denotes no significant difference; * indicates *P* < 0.05; ** indicates *P* < 0.01; *** indicates *P* < 0.001; **** indicates *P* < 0.0001).

To evaluate the functionality of the predicted defense systems within GI*_trmA_* and MGI*_trmE_* in conferring defense against phages, these systems were cloned and assessed in the model organism *E. coli* BW25113 as the sensitive host bacterium. In GI*_trmA_*, a 9.9 kb DNA fragment encompassing Type I RM, KwaAB, and DarTG systems (Sputcn32_3523–3529) was identified to be co-transcribed, suggesting a functional unit. Similarly, within MGI*_trmE_*, a 12.3 kb DNA fragment containing a Type I RM system and RolC (Sputcn32_3981–3587) was also found to be co-transcribed. Subsequently, the 9.9 kb and 12.3 kb DNA fragments from GI*_trmA_* and MGI*_trmE_*, respectively, were cloned into plasmid vector pBBR1Cm and introduced into *E. coli* BW25113, generating BW25113/pGI*_trmA_*Ds and BW25113/pMGI*_trmE_*Ds. These engineered bacteria were then exposed to a range of phages including tailed dsDNA phages T1, T5, EEP, T4, and T7 from the *Siphoviridae*, *Myoviridae,* and *Podoviridae* families. By comparing the phage efficiency of plating (EOP) on system-containing bacteria with control cells, it was observed that pGI*_trmA_*Ds and pMGI*_trmE_*Ds provided significant protection against four phages (T1, EEP, T4, and T7) but not against T5 phage. The presence of pGI*_trmA_*Ds led to a 10-fold to 70-fold reduction in the production of T1, EEP, and T7 phages compared with control cells, whereas pMGI*_trmE_*Ds decreased production by 100-fold to 1,000-fold. Both constructs notably decreased T4 phage production by 10^6^-fold compared with control cells (Fig. 4a and b). These findings indicate the GI*_trmA_* and MGI*_trmE_* function as defense islands, enhancing the defense capabilities of host bacteria against a diverse array of phages.

To explore whether GI*_trmA_* and MGI*_trmE_* restrict plasmid transfer, we tested four plasmids with distinct purposes to assess their defense capabilities against foreign plasmids. These plasmids included pBBR1Cm for constitutive expression of target genes, pMMB207 (containing the Tac promoter) for protein overexpression (20), pMBLCas9 for gene knockout via Cas9 (21, 22), and pSC189, a transposon delivery plasmid for insertional mutagenesis (23) (Table S5).

Since conjugation is the most commonly used method for plasmid transfer in *Shewanella*, we conducted conjugation assays with *E. coli* WM3064 as the donor strain for transferring plasmids, and deletion mutants ΔGI*_trmA_*, ΔMGI*_trmE_*, and ΔCGI48 as recipient strains, with wild-type CN32 as a control. The transfer efficiency was then calculated as the ratio of transconjugants to recipient cells. The results showed that the deletion of GI*_trmA_* or MGI*_trmE_* led to a 10^4^- to 10⁵-fold increase in pBBR1Cm transfer efficiency, respectively reaching 5.8 × 10⁻³ or 9.6 × 10⁻^4^ transconjugants per recipient cell. However, deletion of CGI48 had no significant increase in pBBR1Cm transfer efficiency (Fig. 4c). For the pMMB207 plasmid, removal of GI*_trmA_* resulted in a 1.4 × 10²-fold increase in transfer efficiency, yielding 8.7 × 10⁻⁴ transconjugants per recipient cell, whereas deletion of MGI*_trmE_* caused a 38-fold increase, resulting in 2.3 × 10⁻⁴ transconjugants per recipient cell. Again, no significant effect was observed with the deletion of CGI48 (Fig. 4d).

Given that R-M systems can degrade plasmid DNA prior to homologous recombination, potentially reducing gene knockout efficiency, we also assessed the transfer efficiency of pMBLCas9, a plasmid used for gene deletion via *Streptococcus pyogenes* Cas9. Deletion of either GI*_trmA_* or MGI*_trmE_* led to a 10³-fold increase in pMBLCas9 transfer efficiency, reaching 10⁻⁵ transconjugants per recipient cell, compared with the absence of transconjugants for wild-type CN32 or ΔCGI48 (Fig. 4e). Furthermore, we assessed pSC189, which is used for insertional mutagenesis and high-throughput screening of target genes. Removal of GI*_trmA_* resulted in a 1.9 × 10³-fold increase in pSC189 transfer efficiency, yielding 1.9 × 10⁻⁵ transconjugants per recipient cell, which was significantly higher than the 6.2 × 10⁻⁷ efficiency observed in the MGI*_trmE_* deletion mutant (Fig. 4f).

To examine whether GI*_trmA_* and MGI*_trmE_* act synergistically in plasmid defense, we created a strain lacking both GI*_trmA_* and MGI*_trmE_* (ΔGI*_trmA_* + MGI*_trmE_*). The conjugation efficiency of all these four plasmids into the ΔGI*_trmA_* + MGI*_trmE_* strain was higher than that into the ΔMGI*_trmE_* strain, but there was no significant fold difference compared with the transfer efficiency into the ΔGI*_trmA_* strain (Fig. 4c through f). These findings indicate that both GI*_trmA_* and MGI*_trmE_* contribute to the defense against foreign plasmids, with GI*_trmA_* demonstrating a stronger defense capability. However, the deletion of both systems does not result in a synergistic enhancement of plasmid transfer efficiency in strain CN32.

### The defense systems in GI*_trmA_* and MGI*_trmE_* are widely distributed in gram-negative bacteria

**Fig 5:**
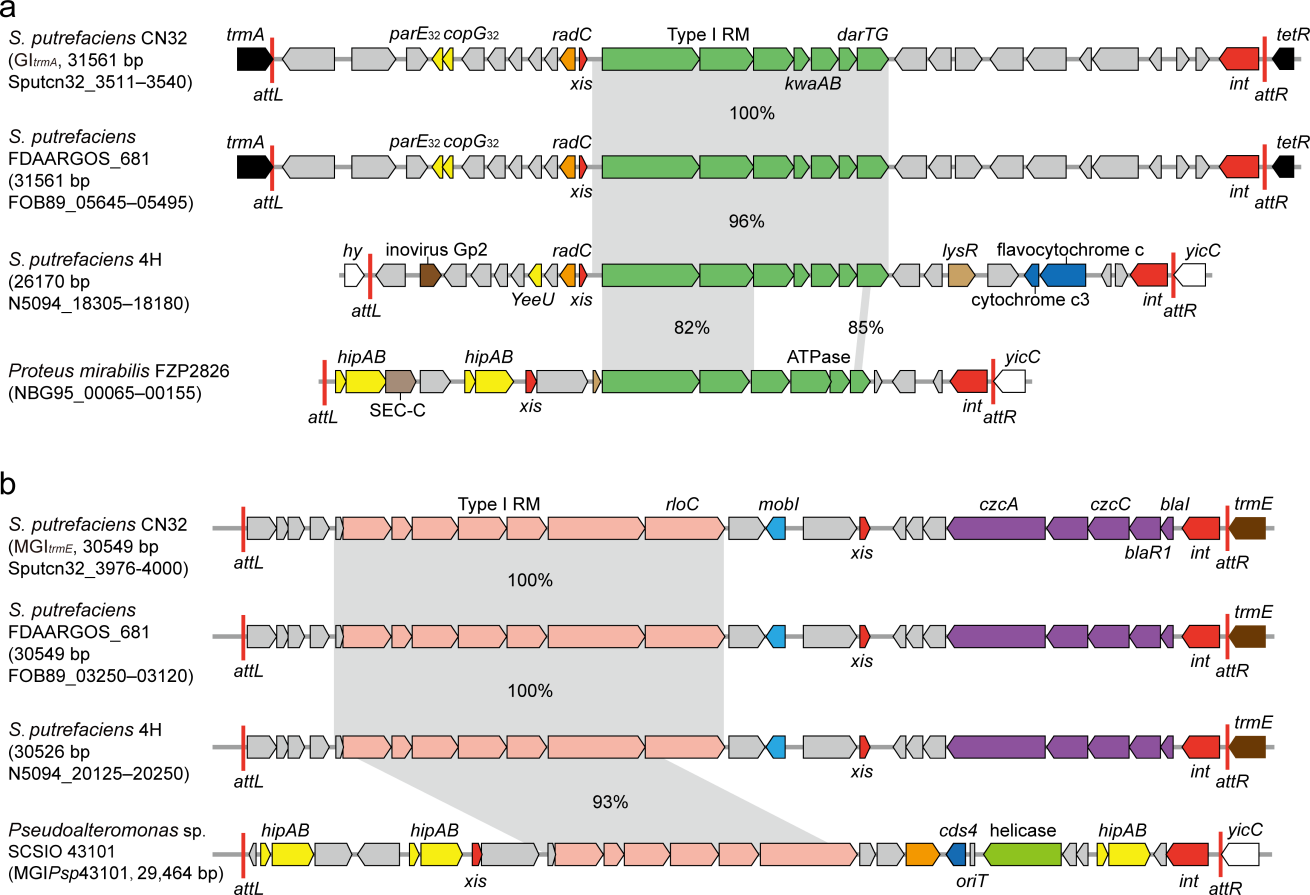
The defense systems in GI*_trmA_* and MGI*_trmE_* are widely distributed in gram-negative bacteria. Sequence comparison of the defense systems in GI*_trmA_* (a) and MGI*_trmE_* (b) with their homologous defense systems in their corresponding genomic islands. The predicted length of the genomic islands and their integration sites was shown. Open reading frames with putative functions are shown in different colors.

To gain insights into the distribution of the defense systems in GI*_trmA_* and MGI*_trmE_*, a search for homologs of GI*_trmA_*Ds and MGI*_trmE_*Ds was conducted in the NCBI database using BLASTN. A total of 19 GI*_trmA_*Ds homologs were identified based on nucleotide sequence (≥40% coverage and ≥70% identity). Among these, four homologs were found in *Shewanella*, seven in *Alteromonas*, and four in *Proteus*. Additionally, homologs were found in strains of *Marinomonas*, *Vibrio*, *Hahella,* and *Pseudoalteromonas*. Further analysis revealed that one of the GI*_trmA_*Ds homologs located on a genomic island in *S. putrefaciens* strain FDAARGOS_681, integrated into *trmA* with 100% identity with GI*_trmA_*. In addition, 10 homologs were located on genomic islands integrated in the *yicC* gene locus in *Shewanella, Alteromonas,* and *Proteus* (Fig. 5a; Table S3).

Similarly, 12 MGI*_trmE_*Ds homologs were also identified based on nucleotide sequence (with ≥60% coverage and ≥80% identity). Among these, six homologs were found in *Shewanella,* whereas others were found in *Pseudoalteromonas*, *Marinomonas*, *Vibrio,* and *Alteromonas* strains. Of these, five homologs were located in the genomic islands integrated into *trmE*, four were in genomic islands integrated into *yicC*, and some were predicted to be located in the genomic islands integrated into the *ssrA* gene and tRNA^Val^ (Table S4). The *S. putrefaciens* strain FDAARGOS_681, isolated from subsurface rock in Cerro Negro, was found to contain both GI*_trmA_* and MGI*_trmE_* with 100% identities (Fig. 5). Interestingly, *S. putrefaciens* 4H harbors a genomic island showing 100% identity with MGI*_trmE_*Ds, and another genomic island containing a defense system displaying 96% identity with GI*_trmA_*Ds. In contrast to CN32, the genomic island containing GI*_trmA_*Ds in *S. putrefaciens* 4H is integrated in *yicC*, not the *trmA* gene (Fig. 5). Overall, these findings indicated that the defense systems in GI*_trmA_* and MGI*_trmE_* are widely distributed in gram-negative bacteria. They function as the cargo genes, coupling with various genomic islands at different integration sites.

## DISCUSSION

Genetic modification of bacteria is crucial for enhancing industrial applications, such as metabolite production and growth rates (24). However, the presence of bacterial defense mechanisms, such as R-M systems and CRISPR, presents significant challenges to genetic manipulation. These systems serve as protective barriers against foreign DNA, complicating the introduction of desired genetic modifications. To circumvent these challenges, various strategies have been developed. One widely utilized method involves the in *vitro* methylation of plasmid DNA, which effectively prevents recognition by R-M systems (25, 26). Additionally, knocking out or suppressing specific R-M and CRISPR loci has proven effective in eliminating these barriers, thereby facilitating successful genetic modifications (11). The emergence of phage-derived anti-CRISPR proteins represents another promising approach, providing temporary inhibition of CRISPR systems to further enhance genetic manipulation efficiency (27). These strategies have significantly improved the success of genetic engineering in recalcitrant bacterial species, particularly where R-M and CRISPR systems have been well characterized. Nevertheless, many bacteria harbor multiple, often uncharacterized defense mechanisms, complicating the landscape of genetic manipulation.

Defense systems are often clustered within mobile genetic elements, forming defense islands (15). In this study, we introduce a novel strategy for targeting these defense islands within bacterial genomes, with a specific focus on *S. putrefaciens* CN32. Our systematic methodology encompasses three critical steps: predicting defense systems using public databases, mapping these systems within the bacterial genome and predicting the boundaries of the associated genomic islands, and executing targeted knockouts of the identified defense islands.

Through this approach, we successfully identified two significant defense islands, GI*_trmA_* and MGI*_trmE_*, in *S. putrefaciens* CN32. The targeted knockout of these islands resulted in a substantial increase in the efficiency of genetic manipulation, highlighting the effectiveness of our strategy. Specifically, we employed an excisionase-based approach, leveraging the unique excisionases and attachment sites associated with these defense islands. By overexpressing the excisionase via a plasmid, we efficiently excised the defense islands from the chromosome at their designated attachment sites, creating strains devoid of these targeted elements.

Importantly, for the defense island like the GI*_trmA_* described in this study, which encodes a functional toxin-antitoxin system, it is crucial to first knock out this system before applying the excisionase-based method. Therefore, for defense islands with toxin-antitoxin systems, we also recommend utilizing the Cas9-NE method, which simplifies the process by leveraging CRISPR/Cas9 technology alongside the natural excision of these islands (22). This approach requires only the expression of a specialized single-guide RNA (sgRNA) targeting the defense island and the Cas9 protein. This straightforward method facilitates the efficient deletion of defense islands, making genetic manipulation more accessible and effective (22). For instances where certain defense islands are non-excisable (with excision efficiency <10^−6^) or lack identifiable excisionases or attachment sites, alternative methods that facilitate the effective removal of these islands as large DNA fragments can be explored.

In conclusion, targeting defense islands provides an effective strategy for developing engineered bacterial strains suitable for genetic manipulation. The prevalence of these islands in various bacterial populations highlights the potential of our approach to advance microbial research and biotechnology. By improving our ability to target these defense mechanisms, we can create new opportunities for biotechnological innovations that contribute to environmental microbiology and sustainable industrial practices.

## MATERIALS AND METHODS

### Bacterial strains and growth conditions

The bacterial strains and plasmids used in this study are listed in Table S5. *Shewanella* and *E. coli* strains were grown in LB medium at 30°C and 37°C, respectively. *E. coli* WM3064 was cultivated in LB supplemented with 0.3 mM DAP (2,6-diamino-pimelic acid). Cm (chloramphenicol, 30 µg mL^−1^) and Kan (kanamycin, 50 µg mL^−1^) were used in *E. coli*, and Cm (10 µg mL^−1^) was used in *Shewanella*. IPTG (isopropyl-*β*-D-thiogalactopyranoside) was used as an inducer.

### Construction of plasmids

The primers used in this study are listed in Table S6. The encoding regions of *xis_3522_* and *xis_3991_* were amplified from CN32 and cloned into the *Eco*RI and *Hind*III sites of pHGECm using T4 ligase, generating pXis_3522_ and pXis_3991_. The encoding regions of *parE*_32_*, copG*_32_, and *copG*_32_-*parE*_32_ were amplified from CN32 and inserted into the *Pst*I and *Hind*III sites of pCA24N, generating pCA24N-*parE*_32_, pCA24N-*copG*_32_, and pCA24N-*copG*_32_-*parE*_32_. To clone the defense systems in GI*_trmA_*, the 9,878 bp GI*_trmA_*Ds region was divided into three overlapping DNA segments, and these DNA segments were produced by PCR amplification and inserted into plasmid pBBR1Cm, generating pGI*_trmA_*Ds. Similarly, the 12,275 bp MGI*_trmE_*Ds region was inserted into pBBR1Cm, producing pMGI*_trmE_*Ds.

### Construction of gene deletion mutants in CN32

The deletion mutant was constructed based on the suicide plasmid pK18*mobsacB*-Cm as described previously (13). Briefly, the upstream and downstream regions of the target gene were amplified from CN32 using the primers listed in Table S6 and inserted into pK18*mobsacB*-Cm using T4 ligase. Then, the pK18*mobsacB*-Cm-derived plasmids were introduced into CN32 via conjugation. After mating, the single crossover mutants were selected using Cm resistance and verified by PCR. Then, the mutants were spread in the LB plates containing 10% sucrose without NaCl and cultured at 30°C for 24–36 h. Finally, the double crossover mutants sensitive to Cm were screened by PCR verification. By using this deletion method, the strain Δ*hns*, with a deletion of 390 bp within the coding region of *h-ns* (*Sputcn32_2512*), and the strain Δ*parE_32_*, with a deletion of the 281 bp region within the coding region of *parE_32_* (*Sputcn32_3514*) were constructed. All the mutants were confirmed by PCR sequencing.

### Location of genomic islands GI*_trmA_* and MGI*_trmE_*

A circular comparison image for strains CN32, W3-18-1, and ANA-3 was generated using BRIG software (28). The locations of the two ends of GI*_trmA_* and MGI*_trmE_* were determined using Mauve (29). The attachment sites of GI*_trmA_* and MGI*_trmE_* were verified by amplification and sequencing. Specifically, the 15wS/15wA primer set was used for ΔGI*_trmA_*, and the 19wS/19wA primer set was used for ΔMGI*_trmE_* (Table S6).

### Quantification of the excision rate of GI*_trmA_* and MGI*_trmE_*

We conducted real-time quantitative PCR (qPCR) assays to quantify the excision rate of genomic islands as previously reported methods (30, 31). For GI*_trmA_* and MGI*_trmE_*, *attB/gyrB* indicated the excision rate of each genomic island after excision, in which *attB* was generated only when the genomic island had been removed, and chromosomal *gyrB* was used as the reference gene. The primers used for the qPCR assays are listed in Table S6. To test the regulation of Xis on the excision of GI*_trmA_* and MGI*_trmE_*, pXis_3522-_ and pXis_3991_-containing strains were induced with 1.0 mM IPTG for 6 h at an OD_600_ of 0.8–1.

### Conjugation assays

The plasmids were transferred from *E. coli* WM3064 into *Shewanella* strains by conjugating assays as described previously. Briefly, the donor and recipient cells were mixed and co-cultured in the LB plate containing DAP. Then, the cells from the lawn were streaked on an LB agar plate with antibiotics to select for transconjugants. The transconjugants were confirmed by PCR verification. Transfer efficiency was calculated as the ratio of transconjugants per recipient cells.

### Phage defense assays

The double-agar layer method was used to determine the defense ability of GI*_trmA_*Ds and MGI*_trmE_*Ds to phage. First, 3 mL of overnight cultures of BW25113/pGI*_trmA_*Ds and BW25113/pMGI*_trmE_*Ds were mixed with 12 mL of top medium (0.8% LB agar medium) supplemented with Cm and plated onto the bottom plate (1.5% LB agar medium), and strain BW25113/pBBR1Cm was used as a control. Then, the purified phages T1, T5, EEP (32), T4, and T7 were diluted and dropped onto the bacterial lawn. Finally, the plaques were visualized after 10–12 h incubation at 37°C and used to calculate the phage titer.

### Statistics and reproducibility

Data analyses were performed using GraphPad Prism (version 7.00). The quantitative data in each experiment were analyzed by unpaired *t*-tests for two samples. Asterisks represent statistically significant differences (ns, (not significant); *P < 0.05; **P < 0.01; ***P < 0.001; ****P < 0.0001). Individual data points are plotted with lines at the mean, and error bars represent the standard deviation in each figure. Biological replicates are shown in the figure legends.

## Data Availability

The complete genome sequences of strains CN32, W3-18-1, and ANA-3 are available in GenBank under accession numbers CP000681, CP000503, and NC_008577, respectively. All sequence data for plasmids generated in this study can be freely accessed via the Science Data Bank at https://doi.org/10.57760/sciencedb.20715.

## SUPPLEMENTAL MATERIAL

Supplemental material aem.02499-24-s0001.pdf Figure S1; Tables S1 to S6.

ASM does not own the copyrights to Supplemental Material that may be linked to, or accessed through, an article. The authors have granted ASM a non-exclusive, world-wide license to publish the Supplemental Material files. Please contact the corresponding author directly for reuse.
