# Supplementary material for: Efficient genetic manipulation of Shewanella through targeting defense islands
Source: Appl Environ Microbiol. 2025 Mar 21;91(4):e02499-24. doi: 10.1128/aem.02499-24 (PMC12016545; doi:10.1128/aem.02499-24)
Supplement: Supplemental material — Figure S1; Tables S1 to S6. [file aem.02499-24-s0001.pdf]

SUPPLEMENTARY FILE

Efficient genetic manipulation of *Shewanella* through targeting defense islands

Yilong Ruan[1,2,3], Huan Tang[1,2,3], Tongxuan Cai[1,4], Xiaofei Du[1,2,3], Tianlang Liu[1,2,3], Xiaoxue Wang[1,2,3], Pengxia Wang[1,2,3*]

*To whom correspondence should be addressed. Pengxia Wang, E-mail: wangpengxia@scsio.ac.cn

This PDF file includes:

Figure S1

Tables S1 to S6

References

Figure S1. ParE32 and CopG32 in GI*trmA* constitute a toxin-antitoxin pair. (**a**) Sequence alignment of GI*trmA*-encoded ParE32/CopG32 and CP4So-encoded ParEso/CopGso. Cell growth (**b**) and cell viability (**c**) of cells overexpressing ParE32, CopG32, and ParE32/CopG32 in *E. coli* BW25113. (**d**) Growth of BW25113 cells overexpressing ParE32, CopG32, and ParE32/CopG32 on LB plates with and without 0.5 mM isopropyl-β-D-thiogalactopyranoside (IPTG). (**e**) Colony-forming units (CFU) of strain BW25113 over-expressing ParE32, CopG32, and ParE32/CopG32 on LB plates with chloramphenicol.

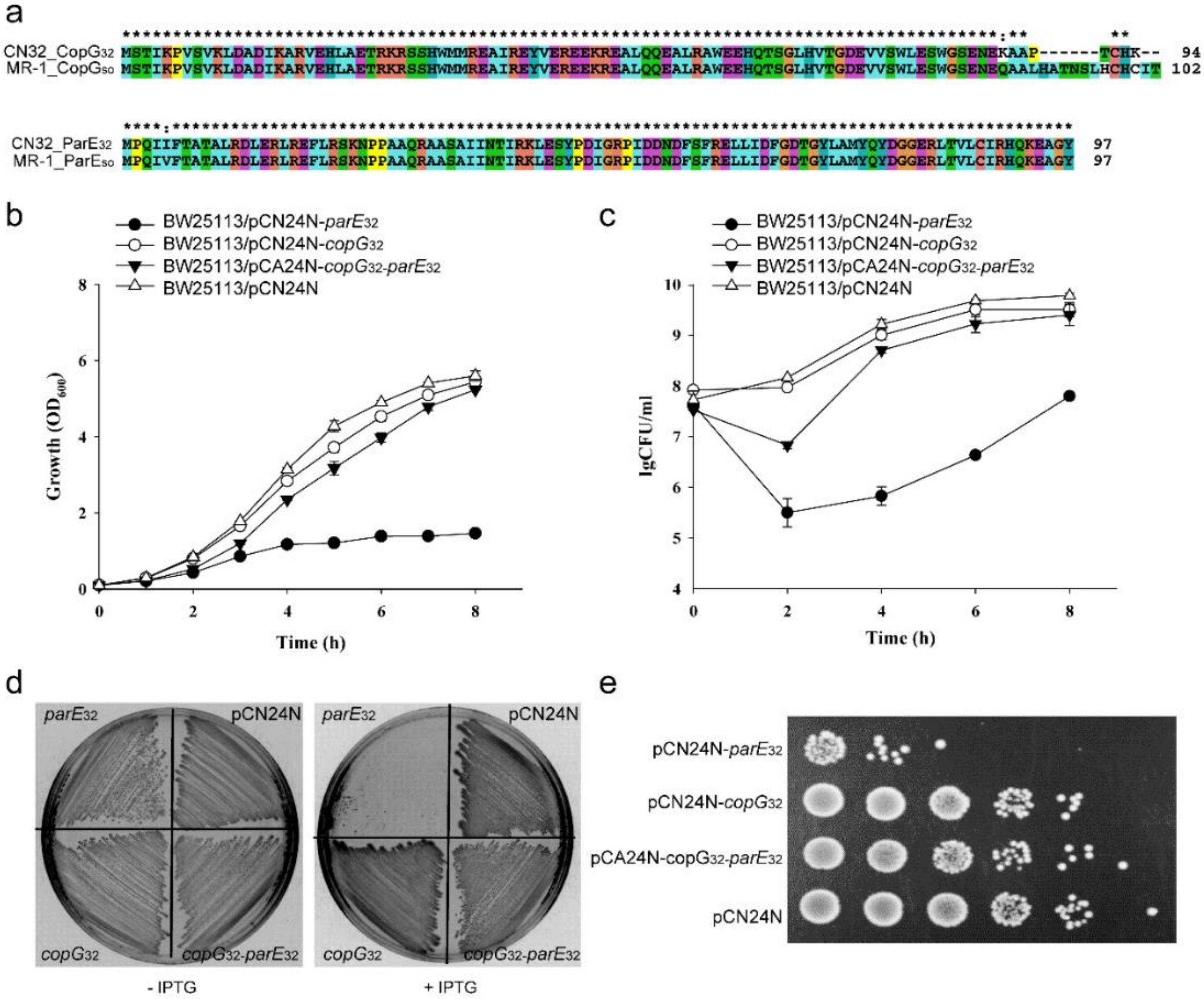

Table S1. Sequence analysis of genomic island GI$_{trmA}$.

Locus_tag	Start	End	Strand	Functions
Sputcn32_3510	4082595	4083695	+	tRNA (uracil-5-)-methyltransferase TrmA
attL	4083648	4083673	+	left attachment site
Sputcn32_3511	4085671	4084088	-	transposase and inactivated derivatives-like protein
Sputcn32_3512	4086196	4087656	+	hypothetical protein
Sputcn32_3513	4087970	4088638	+	transposase
Sputcn32_3514, ParE$_{32}$	4089118	4088825	-	plasmid stabilization system
Sputcn32_3515, CopG$_{32}$	4089390	4089106	-	transcriptional regulator, CopG family
Sputcn32_3516	4090399	4089677	-	retinol acyltransferase domain protein
Sputcn32_3517	4091111	4090545	-	phage integrase family protein
Sputcn32_3518	4091641	4091228	-	conserved hypothetical protein
Sputcn32_3519	4092220	4091846	-	YagBYeeUYfjZ family protein
Sputcn32_3520	4092722	4092360	-	conserved hypothetical protein
Sputcn32_3521	4093269	4092763	-	DNA repair protein RadC
Sputcn32_3522, Xis$_{3522}$	4093402	4093599	+	phage transcriptional regulator AlpA, excisionase
Sputcn32_3523	4094178	4097291	+	protein of unknown function DUF450, type III restriction enzyme, res subunit
Sputcn32_3524	4097340	4098989	+	N-6 DNA methylase
Sputcn32_3525	4098979	4100241	+	restriction modification system DNA specificity domain
Sputcn32_3526	4100238	4100795	+	conserved hypothetical protein, predicted KwaA
Sputcn32_3527	4100798	4101634	+	conserved hypothetical protein, predicted KwaB_2
Sputcn32_3528	4101638	4102276	+	conserved hypothetical protein, predicted DarT
Sputcn32_3529	4102290	4103330	+	macro domain-containing protein, predicted DarG
Sputcn32_3530	4104458	4103478	-	alcohol dehydrogenase, zinc-binding domain protein
Sputcn32_3531	4105255	4104548	-	putative transcriptional regulator, TetR family
Sputcn32_3532	4105327	4106193	+	transcriptional regulator, LysR family
Sputcn32_3533	4107601	4106474	-	conserved hypothetical protein
Sputcn32_3534	4108939	4107695	-	conserved hypothetical protein
Sputcn32_3535	4109687	4109328	-	tetraheme cytochrome c
Sputcn32_3536	4111236	4109716	-	flavocytochrome c
Sputcn32_3537	4111902	4111579	-	hypothetical protein
Sputcn32_3538	4112422	4112742	+	transcriptional regulator, XRE family
Sputcn32_3539	4113074	4113436	+	conserved hypothetical protein
Sputcn32_3540	4115041	4113779	-	phage integrase family protein
attR	4115209	4115234	+	right attachment site
Sputcn32_3541	4115321	4115935	-	transcriptional regulator, TetR family

The red font showed the genes encoding the defense system GI$_{trmA}$Ds.

Table S2. Sequence analysis of genomic island MGI$_{trmE}$.

Locus_tag	Start	End	Strand	Functions
attL	4624490	4624563	+	left attachment site
Sputcn32_3976	4624844	4625686	+	hypothetical protein
Sputcn32_3977	4625683	4626114	+	hypothetical protein
Sputcn32_3978	4626111	4626623	+	hypothetical protein
Sputcn32_3979	4626756	4627382	+	Resolvase, N-terminal domain
Sputcn32_3980	4627574	4627804	+	transcriptional regulator
Sputcn32_3981	4627801	4629300	+	N-6 DNA methylase
Sputcn32_3982	4629297	4629926	+	conserved hypothetical protein
Sputcn32_3983	4629919	4631373	+	putative transcriptional regulator
Sputcn32_3984	4631370	4632887	+	N-6 DNA methylase
Sputcn32_3985	4632887	4634092	+	restriction modification system DNA specificity domain
Sputcn32_3986	4634093	4637155	+	type I site-specific deoxyribonuclease, HsdR family
Sputcn32_3987	4637195	4639606	+	conserved hypothetical protein, RloC
Sputcn32_3988	4639658	4640692	+	putative cytoplasmic protein
Sputcn32_3989	4641334	4640750	-	hypothetical protein
Sputcn32_3990	4642062	4643732	+	superfamily II helicase and inactivated derivatives-like protein
Sputcn32_3991, Xis$_{3991}$	4643792	4644136	+	phage transcriptional regulator AlpA, excisionase
Sputcn32_3992	4645162	4644830	-	conserved hypothetical protein
Sputcn32_3993	4645718	4645185	-	conserved hypothetical protein
Sputcn32_3994	4646434	4645739	-	conserved hypothetical protein
Sputcn32_3995	4649567	4646424	-	heavy metal efflux pump, CzcA family
Sputcn32_3996	4650823	4649579	-	secretion protein HlyD
Sputcn32_3997	4652115	4650832	-	outer membrane efflux protein
Sputcn32_3998	4653109	4652129	-	peptidase M56, BlaR1
Sputcn32_3999	4653495	4653118	-	transcriptional regulator, TrmB
Sputcn32_4000	4654931	4653762	-	phage integrase family protein
attR	4655039	4655112	+	right attachment site
Sputcn32_4001	4656456	4655095		tRNA modification GTPase TrmE

The red font showed the genes encoding the defense system MGI$_{trmE}$Ds.

Table S3. Distribution of 9878-bp GI$_{trmA}$Ds region in gram-negative bacteria.

Bacterial strains	Accession	Coverage	Identity	homologous protein	predicted integration site
Shewanella putrefaciens FDAARGOS_681	CP046329.1	100%	100	FOB89_05585–05555	*trmA*
Shewanella putrefaciens 4H	CP104755.1	94%	95.85	N5094_18255–18225	*yicC*
Shewanella xiamenensis HD6416	CP079718.1	86%	87.63	KXJ80_04490–04460	*yicC*
Alteromonas pelagimontana 5.12	CP052766.1	70%	81.75	CA267_009100–009180	*yicC*
Marinomonas sp. CT5	CP025572.1	50%	82.23	C0J08_03230–03220	undetermined
Alteromonas mediterranea U8	CP004852.1	48%	82.1	I634_19150–19145	*yicC*
Alteromonas mediterranea U7	CP004851.1	48%	82.17	I876_19400–19405	*yicC*
Alteromonas mediterranea U4	CP004849.1	48%	82.17	I607_19025–19030	*yicC*
Shewanella marisflavi ECSMB14101	CP041153.1	50%	81.94	FGA12_18270–17235	undetermined
Proteus mirabilis FZP2826	CP098446.1	48%	81.9	NBG95_00110–00115	*yicC*
Proteus mirabilis NYP6	CP110375.1	48%	81.88	ONR69_00100–00105	*yicC*
Proteus mirabilis NYP73	CP110377.1	48%	81.88	ONR69_00100–00105	*yicC*
Proteus mirabilis NYP69	CP110376.1	48%	81.88	ONR68_00100–00105	*yicC*
Vibrio rumoiensis FERM P-14531	AP018685.1	50%	81.77	(unannotated)	undetermined
Alteromonas macleodii EZ55	OX359237.1	50%	81.39	EZ55_00008–00010	undetermined
Alteromonas sp. KC14	AP024236.1	48%	80.97	KUC14_28800–28810	undetermined
Alteromonas sp. KC3	AP024235.1	48%	80.97	KUC3_29030–29040	undetermined
Hahella sp. KA22	CP035490.1	48%	80.86	EUZ85_30300–30305	undetermined
Pseudoalteromonas phenolica KCTC 12086	CP013187.1	43%	73.91	PP2015_444–445	undetermined

Table S4. Distribution of 12275-bp MGI$_{trmE}$Ds region in gram-negative bacteria.

Description	Accession	Coverage	Identity (%)	homologous protein	predicted integration site
Shewanella putrefaciens FDAARGOS_681	CP046329.1	100%	100	FOB89_03225–03195	*trmE*
Shewanella putrefaciens 4H	CP104755.1	100%	100	N5094_20180–20145	*trmE*
Pseudoalteromonas sp. SCSIO 43101	CP072673.1	71%	93.33	J8Z25_02900–02875	*yicC*
Marinomonas profundi M1K-6	CP073013.1	88%	94.15	H9K44_14500–14510	$tRNA^{Val}$
Shewanella chilikensis DC57	CP045857.1	68%	93.98	GII14_19940–19970	*yicC*
Vibrio parahaemolyticus DLM1799	CP064035.1	68%	93.53	H9K44_14530–14500	*yicC*
Vibrio ostreae OG9-811	CP076643.1	66%	94.22	KNV97_18735–18765	undetermined
Alteromonas macleodii str. 'Balearic Sea AD45'	CP003873.1	85%	93.75	AMBAS45_19685–19720	*yicC*
Shewanella baltica NCTC10737	LR134321.1	72%	84.61	NCTC10737_04347–04353	*trmE*
Aeromonas veronii jsyz553301	CP106828.1	66%	86.17	NBH81_07145–07170	*ssrA*
Shewanella baltica 11FHM2	CP051529.1	88%	81.79	HHE93_21900–21935	*trmE*
Shewanella sp. DAU305	CP113808.1	63%	84.77	OX890_02040–02070	*trmE*

Table S5. Strains and plasmids used in this study.

Strains/plasmids	Description[a]	Reference
***Shewanella putrefaciens* strains**		
CN32	*Shewanella putrefaciens* CN32 wild type， isolated from anaerobic shale sandstone at the depth of 250 m in the Morrison formation of Cerro Negro, New Mexico	(1)
Δ*hns*	Deletion mutant of *h-ns* gene (Sputcn32_2512) in CN32	This study
Δ$parE_{32}$	Deletion mutant of $parE_{32}$ gene from GI_{trmA} in CN32	This study
ΔGI_{trmA}	Deletion mutant of GI_{trmA} in CN32	This study
ΔMGI_{trmE}	Deletion mutant of MGI_{trmE} in CN32	This study
ΔGI_{trmA}+MGI_{trmE}	Deletion mutant of GI_{trmA} and MGI_{trmE} in CN32	This study
ΔCGI48	Deletion mutant of CGI48 in CN32	(2)
***Escherichia coli* strains**		
WM3064	RP4(tra) in chromosome, DAP-, 37°C	(3)
K-12 BW25113	$lacI^q$ $rrnB_{T14}$ Δ$lacZ_{WJ16}$ *hsdR*514 ΔaraBAD_{AH33} ΔrhaBAD_{LD78}	(4)
**Plasmids**		
pBBR1Cm	Cm^R; broad-host-range expression plasmid	(5)
pGI_{trmA}Ds	Cm^R; Sputcn32_3523-3529 cloned into pBBR1Cm	This study
pMGI_{trmE}Ds	Cm^R; Sputcn32_3981-3987 cloned into pBBR1Cm	This study
pMMB207	Cm^R, RSF1010 derivative, *IncQ* $lacI^q$ Tac *ori*T	(6)
pMBLCas9	Cm^R, Cas9 expressing and target sgRNAs cloning for gene deletion	(7, 8)
pSC189	Km^R, Cm^R, The *Himar1* suicide transposon delivery vector	(9)
pCA24N	Cm^R; $lacI^q$, IPTG inducible expression plasmid in *E. coli*	(10)
pCA24N-$ParE_{32}$	Cm^R; $lacI^q$, $P_{T5\text{-}lac}$::$parE_{32}$	This study
pCA24N-$copG_{32}$	Cm^R; $lacI^q$, $P_{T5\text{-}lac}$::$copG_{32}$	This study
pCA24N-$copG_{32}$-$parE_{32}$	Cm^R; $lacI^q$, $P_{T5\text{-}lac}$::$copG_{32}$-$parE_{32}$	This study
pHGECm	Cm^R; Kan^R; IPTG inducible expression plasmid	(11)
$pXis_{3522}$	Cm^R, expression plasmid for Sputcn32_3522 from GI_{trmA}	This study
$pXis_{3991}$	Cm^R, expression plasmid for Sputcn32_3991 from MGI_{trmE}	This study
pK18*mobsacB*-Cm	Km^R, Cm^R, *SacB*, suicide plasmid used for gene knockout	(12)
pK18Cm-*hns*	pK18*mobsacB*-Cm containing the homologous arms of *h-ns*	This study
pK18Cm-$parE_{32}$	pK18*mobsacB*-Cm containing the homologous arms of $parE_{32}$	This study

[a]Cm^R, chloramphenicol resistance; Kan^R, kanamycin resistance. The sequence of plasmids constructed in this study can be freely accessed via the Science Data Bank at https://doi.org/10.57760/sciencedb.20715.

Table S6. Primers used in this study.

Primers	Sequence (5′-3′)	Purpose
**Plasmid construction**		
Xis$_{3522}$-F	CGGAATTCATGAAACTAATTCGCCTTAC	pXis$_{3522}$
Xis$_{3522}$-R	CCCAAGCTTTTAAACGGTTGCACTTTGGGC	
Xis$_{3991}$-F	CGGAATTCATGACTGAAAAAGAGATGCTTCAG	pXis$_{3991}$
Xis$_{3991}$-R	CCCAAGCTTCTAATATCTCCGGCCATACAAC	
3514-PstI-S	TCGACTGCAGCCACAAATAATCTTCACTGC	pCA24N-*parE*$_{32}$
3514-HindIII-A	CCCAAGCTTTCAGTAACCGGCTTCTTTTTG	
3515-PstI-S	TCGACTGCAGAGCACAATCAAACCCGTGTC	pCA24N-*copG*$_{32}$
3515-HindIII-A	CCCAAGCTTTTATTTGTGGCATGTAGGGGC	
3515-PstI-S	TCGACTGCAGAGCACAATCAAACCCGTGTC	pCA24N-*copG*$_{32}$-*parE*$_{32}$
3514-HindIII-A	CCCAAGCTTTCAGTAACCGGCTTCTTTTTG	
**Construction of pGI$_{trmA}$Ds**		
GI$_{trmA}$-1-F	GTGACCGTGTGCTTCGAATTCGCTCATACCAACACCCGTTTTAC	pGI$_{trmA}$Ds
GI$_{trmA}$-1-R	CTAAGCGCTACCAAGATTGGCTACCAGTATTAAA	
GI$_{trmA}$-2-F	CCAATCTTGGTAGCGCTTAGTTAGTCGTGCCAGTATC	
GI$_{trmA}$-2-R	TGATCCATCTTTATCCTCGCTCTATGAAGGTGTAAT	
GI$_{trmA}$-3-F	GCGAGGATAAAGATGGATCAAATATTGGCCAGA	
GI$_{trmA}$-3-R	AGGGAACAAAAGCTGGGTACCTTTAATACTATTTCGTTAGCGTTAGGTTT	
**Construction of pMGI$_{trmE}$Ds**		
MGI$_{trmE}$-1-F	GTGACCGTGTGCTTCGAATTCTACCACGACTTTCTGCCGTATCA	pMGI$_{trmE}$Ds
MGI$_{trmE}$-1-R	AATGCGCTCATGATGTCTTCTTATCGCTTAGTTG	
MGI$_{trmE}$-2-F	GAAGACATCATGAGCGCATTAAATAAGAAAAAATTAGAGG	
MGI$_{trmE}$-2-R	GGCTGTCTTGCTCGATATGTTGGCCGAGTTTT	
MGI$_{trmE}$-3-F	ACATATCGAGCAAGACAGCCGTGGCATTTT	
MGI$_{trmE}$-3-R	AGGGAACAAAAGCTGGGTACCAAGTTCAGCCTCGTACACTACTGGT	
**Primers used in determination of the deletion mutants ΔGI$_{trmA}$, MGI$_{trmE}$ and ΔGI$_{trmA}$+MGI$_{trmE}$**		
15wS	GCAGATAGAGAATAGCTTTACC	ΔGI$_{trmA}$
15wA	GGAAGTGCTTGATTCTAATCC	
15dS	CAAGTCATTGTGCCTGTCG	
15dA	GAGCAGCCCTATAGATTTAG	
Int2-F	GTAATAGCCGAATATTGGCAGC	
Int2-R	GAGCAGCCCTATAGATTTAG	
19wS	CAGGCCATTATCTATTAAGTC	ΔMGI$_{trmE}$, ΔGI$_{trmA}$+MGI$_{trmE}$
19wA	CTGCACTTAAAGTCACTTATGG	
19dS	GATTCAGCGGTTCGTAAGAG	
19dA	CTTCCATGACAAAACCCGAC	
Int3-F	TCTCTGATGATTCGGTCTCG	
Int3-R	TGCCAAACATGGCTTGGATG	
**Primers used in construction of Δ*hns***		
H-NS_up-S	ACATGCATGCGTAAATTCAGCGTCTGCATAC	Δ*hns*
H-NS_up-A	CGGAATTCCATATCTATTTTCGTCCCAC	
H-NS_down-S	CGGAATTCTCCATACAAACTCGCAGTAAAAGG	
H-NS_down-A	AGCGTCGACGGTAAATCACTTCGTTAGCC	
H-NS_wS	GTCACGCCTAAATCTGCGAC	
H-NS_wA	CTGCCCATTAATGAACTGGG	
H-NS_dA	CCTTTTACTGCGAGTTTGTATGG	
H-NS_dS	GTGGGACGAAAATAGATATG	
**Primers used in construction of Δ*parE*$_{32}$**		
parE-Up-S	ACATGCATGCACCACAGCTTAACTTTCGCG	Δ*parE*$_{32}$
parE-Up-A	CCGGAATTCAACGGCTGAATCAGTGACGG	
parE-down-S	CCGGAATTCTTATTTGTGGCATGTAGGGG	
parE-down-A	AGCGTCGACGAGCACTTCGTCAATGAATG	

parE-wS	GGCTTCAAAGTTTATCCTAGG	
parE-wA	GTGATTCACTTATCTAAGCAG	
parE-dS	GTAATCCGTCACTGATTCAG	
parE-dA	GTACCACGTTATGCTAAGG	
**Primers used in qPCR to determine the excision rate of GI$_{trmA}$ and MGI$_{trmE}$ in CN32**		
GI$_{trmA}$-qF	TGCAAGGTTACGAGCGGATT	GI$_{trmA}$
GI$_{trmA}$-qR	GGTTCGAGGTCAATGGTTAACG	
MGI$_{trmE}$-qF	CACACGTGGCGCAAGAATC	MGI$_{trmE}$
MGI$_{trmE}$-qR	TCTGAAATCACAGGCCGTTTT	
CN32gyrB-qF	TTCGTACTTTGCTGTTGACCTTCT	Reference gene
CN32gyrB-qR	CTACGGTGCCATCCAATGCT	

Reference:

1. Fredrickson JK, Romine MF, Beliaev AS, Auchtung JM, Driscoll ME, Gardner TS, Nealson KH, Osterman AL, Pinchuk G, Reed JL, Rodionov DA, Rodrigues JL, Saffarini DA, Serres MH, Spormann AM, Zhulin IB, Tiedje JM. 2008. Towards environmental systems biology of *Shewanella*. Nat Rev Microbiol 6:592-603.
2. Zhao Y, Wang W, Yao J, Wang X, Liu D, Wang P. 2022. The HipAB toxin-antitoxin system stabilizes a composite genomic island in *Shewanella putrefaciens* CN-32. Front Microbiol 13:858857.
3. Dehio C, Meyer M. 1997. Maintenance of broad-host-range incompatibility group P and group Q plasmids and transposition of Tn*5* in *Bartonella henselae* following conjugal plasmid transfer from *Escherichia coli*. J Bacteriol 179:538-540.
4. Baba T, Ara T, Hasegawa M, Takai Y, Okumura Y, Baba M, Datsenko KA, Tomita M, Wanner BL, Mori H. 2006. Construction of *Escherichia coli* K-12 in-frame, single-gene knockout mutants: the Keio collection. Mol Syst Biol 2:2006.0008.
5. Zeng Z, Guo XP, Li B, Wang P, Cai X, Tian X, Zhang S, Yang JL, Wang X. 2015. Characterization of self-generated variants in *Pseudoalteromonas lipolytica* biofilm with increased antifouling activities. Appl Microbiol Biotechnol 99:10127-10139.
6. Morales VM, Backman A, Bagdasarian M. 1991. A series of wide-host-range low-copy-number vectors that allow direct screening for recombinants. Gene 97:39-47.
7. Wang P, He D, Li B, Guo Y, Wang W, Luo X, Zhao X, Wang X. 2019. Eliminating *mcr-1*-harbouring plasmids in clinical isolates using the CRISPR/Cas9 system. J Antimicrob Chemother 74:2559-2565.
8. Wang P, Du X, Zhao Y, Wang W, Cai T, Tang K, Wang X. 2024. Combining CRISPR/Cas9 and natural excision for the precise and complete removal of mobile genetic elements in bacteria. Appl Environ Microbiol 90:e0009524.
9. Yamaichi Y, Chao MC, Sasabe J, Clark L, Davis BM, Yamamoto N, Mori H, Kurokawa K, Waldor MK. 2015. High-resolution genetic analysis of the requirements for horizontal transmission of the ESBL plasmid from *Escherichia coli* O104:H4. Nucleic Acids Res 43:348-360.
10. Kitagawa M, Ara T, Arifuzzaman M, Ioka-Nakamichi T, Inamoto E, Toyonaga H, Mori H. 2005. Complete set of ORF clones of Escherichia coli ASKA library (a complete set of E. coli K-12 ORF archive): unique resources for biological research. DNA Res 12:291-299.
11. Wang P, Zeng Z, Wang W, Wen Z, Li J, Wang X. 2017. Dissemination and loss of a biofilm-related genomic island in marine *Pseudoalteromonas* mediated by integrative and conjugative elements. Environ Microbiol 19:4620-4637.
12. Wang P, Yu Z, Li B, Cai X, Zeng Z, Chen X, Wang X. 2015. Development of an efficient conjugation-based genetic manipulation system for *Pseudoalteromonas*. Microb Cell Fact 14:11.
